# The Relationship between Functional Inhibition and Binding for K_Ca_2 Channel Blockers

**DOI:** 10.1371/journal.pone.0073328

**Published:** 2013-09-10

**Authors:** David Charles Hammond Benton, Monique Garbarg, Guy William John Moss

**Affiliations:** Department of Neuroscience, Physiology and Pharmacology, University College London, London, United Kingdom; University of Milan, Italy

## Abstract

Small conductance calcium-activated potassium channels (K_Ca_2.1,2.2,2.3) are blocked with high affinity by both peptide toxins (e.g. apamin) and small molecule blockers (e.g. UCL 1848). In electrophysiological experiments, apamin shows subtype selectivity with IC_50_s of ∼100 pM and ∼1 nM for block K_Ca_2.2 and K_Ca_2.3 respectively. In binding studies, however, apamin appears not to discriminate between K_Ca_2.2 and 2.3 and is reported to have a significantly higher (∼20–200-fold) affinity (∼5 pM). This discrepancy between binding and block has been suggested to reflect an unusual mode of action of apamin. However, these binding and electrophysiological block experiments have not been conducted in the same ionic conditions, so it is also possible that the discrepancy arises simply because of differences in experimental conditions. We have now examined this latter possibility. Thus, we measured ^125^I-apamin binding to intact HEK 293 cells expressing K_Ca_2 channels under the same ionic conditions (i.e. normal physiological conditions) that we also used for current block measurements. We find that binding and block experiments agree well if the same ionic conditions are used. Further, the binding of apamin and other blockers showed subtype selectivity when measured in normal physiological solutions (e.g.^125^I-apamin bound to K_Ca_2.2 with *K*_L_ 91±40 pM and to K_Ca_2.3 with *K*_L_ 711±126 pM, while inhibiting K_Ca_2.2 current at IC_50_ 103±2 pM). We also examined K_Ca_2 channel block in Ca^2+^ and Mg^2+^ free solutions that mimic conditions reported in the literature for binding experiments. Under these (non-physiological) conditions the IC_50_ for apamin block of K_Ca_2.2 was reduced to 20±3 pM. Our results therefore suggest that the apparent discrepancy between blocking and binding reported in the literature can be largely accounted for by the use of non-physiological ionic conditions in binding experiments.

## Introduction

Small conductance Ca^2+^-activated potassium channels (SK or K_Ca_2) are widely expressed in vertebrates and have a role in the function of both excitable and inexcitable tissues [1], [2]. Native K_Ca_2 channels were first defined by their sensitivity to intracellular Ca^2+^, low unitary conductance (5–10 pS), lack of voltage dependence and sensitivity to block by the bee venom toxin apamin [3], [4]. Apamin is an 18 amino acid peptide that has since been used in many functional studies and also, in its mono-iodinated form, as a radio-labelled ligand [see e.g. 5,6]. A number of other highly potent and selective toxins have also been identified that target K_Ca_2 channels [7] as well as several potent small molecule inhibitors such as UCL1684 [8] and UCL1848 ([9],see [10] for an excellent review).

Cloning studies have shown that K_Ca_2 channels are encoded by a family of three genes (KCNN1-3) each of which forms a channel alpha subunit (SK1-3 or K_Ca_2.1, 2.2, 2.3) [11]. Functional channels are comprised of four alpha subunits each of which constitutively binds calmodulin, which is responsible for channel gating by Ca^2+^ [12]. Although there is a high degree of sequence identity between all three members of this family, they show important functional differences. In fact, while the rat and human K_Ca_2.2 and K_Ca_2.3 subunits can form functional homomeric channels, only the human (and not the rat) isoform of K_Ca_2.1 is able to do so. Indeed, in both rat and mouse, functional expression of K_Ca_2.1 channels appears to rely on co-assembly with K_Ca_2.2 subunits, via the formation of functional heteromeric channels [13]. Another notable difference between the three K_Ca_2 channel subunits lies in the susceptibility of the channels they form to block by peptide toxins and small molecule inhibitors. For example, apamin has been reported to block the current carried by K_Ca_2.2 channels with an IC_50_ of ≈100 pM while K_Ca_2.3 channels were less sensitive (IC_50_ ≈1 nM) [11]. Human K_Ca_2.1 channels, when expressed in mammalian cell lines, are even less sensitive to apamin with an IC_50_ of ∼3–12 nM [14], [15]. However, a different picture emerges from some direct studies of the binding of ^125^I-apamin to heterologously expressed K_Ca_2 channels. These suggest that apamin binds to all the K_Ca_2 subtypes with very high affinity (in the low picomolar range) and shows a much smaller degree of selectivity. For example, Finlayson *et al*. [16] showed that in saturation binding experiments ^125^I-apamin bound with *K*_L_ values of 6 pM, 8 pM and 270 pM for K_Ca_2.2, K_Ca_2.3 and K_Ca_2.1 respectively. Similarly, Lamy *et al*. [17] reported a value of 6 pM for both K_Ca_2.2 and K_Ca_2.3. Thus, in these experiments, not only did apamin fail to show appreciable selectivity between K_Ca_2.2 and K_Ca_2.3, as seen in functional studies, but the absolute affinity of apamin for all subtypes was much higher (∼20–200-fold) than would have been expected from the concentrations observed to block K_Ca_2 channels in intact cells. One suggestion is that these differences reflect the complex mechanism of action of apamin, a view that has quickly gained favour (see Adelman *et al*. [1] for a review). However, it is possible that such discrepancies simply reflect differing experimental conditions. The aim of the present work was to examine this second possibility by comparing K_Ca_2 channel binding and functional inhibition under near-identical experimental conditions.

## Methods

### Radio-ligand binding studies with ^125^I-apamin

Radio-ligand binding studies were conducted using HEK 293 cell lines stably expressing K_Ca_2.2 or K_Ca_2.3. The cells were cultured in DMEM supplemented with 10% foetal calf serum, 2 mM L-glutamine, penicillin (200 units ml^–1^) and streptomycin (100 µg ml^–1^) in T500 flasks (Nunc). When confluent, these cells were harvested mechanically (to avoid the use of trypsin) into Ca^2+^/Mg^2+^ free HBSS. The cells were centrifuged at 50 g for 2 min, resuspended in DMEM at a density of approximately 2.5×10^6^ cells ml^–1^ and stored at 4°C until used (<2 hr). Cell density was estimated using a haemocytometer.

Routinely, incubations were performed in a total volume of 250 µl comprising 100 µl cell suspension (∼250000 cells), 100 µl ^125^I-apamin and 50 µl displacing agent or incubation medium. The incubation medium contained (in mM) NaCl 140, KCl 5, MgCl_2_ 1, CaCl_2_ 2, glucose 10 and HEPES 10. The pH was adjusted to 7.4 with 1 M NaOH. Non-specific binding was estimated in the presence of 100 nM UCL 1848, a potent K_Ca_2 channel blocker which causes maximal inhibition at this concentration (see Hosseini *et al*. [18], Benton *et al*.[13]). Measurements for each test were performed in triplicate. Separation of cells from unbound ligand was achieved by rapid filtration through Whatman GF/B filters pre-treated with 0.3% v/v polyethyleneimine using a Skatron AS harvester. The quantity of labelled apamin bound was measured using a calibrated γ counter (LKB 1275) and expressed as fmol label/10^6^ cells. All binding experiments were conducted at room temperature (20–25°C).

### Saturation binding experiments

In order to establish a suitable incubation period for equilibrium binding studies we measured the time course of association of ^125^I-apamin to K_Ca_2.2 and K_Ca_2.3 expressing HEK cells by incubating the cells with a low concentration of ^125^I-apamin (20 pM and 60 pM for K_Ca_2.2 and K_Ca_2.3 respectively). This established a 10 minute incubation as appropriate (see results). In order to estimate the maximum total (specific) binding (*B*_max_) and the equilibrium dissociation constant (*K*_L_) the data for total binding was fitted to an equation of the form:
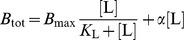
where *B*_tot_ is the total label bound, [L] is the concentration of free ligand and ‘*α*’ is a constant associated with non-specific binding (*B*_ns_), obtained by simultaneously fitting the data for non-specific binding to a straight line:



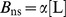



### Competition binding experiments

The ability of test compounds to inhibit ^125^I-apamin binding was measured in the presence of 20–60 pM ^125^I-apamin with or without the test compound. In every experiment each concentration of inhibitor was tested in triplicate and the data presented represents the mean of at least two separate experiments. Inhibition curves were fitted by a variant of Hill-Langmuir equation:
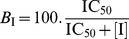
where *B*_I_ is the specific binding of label in the presence of inhibitor as a percentage of the binding in its absence. Estimates of *K*_i_ were obtained using the Cheng-Prussoff correction taking estimates of *K*_L_ from the saturation binding studies.

### Electrophysiology

For electrophysiology, wild type and stably transfected HEK cells were plated in 35 mm dishes. In most experiments K_Ca_2.2 was transiently expressed in HEK 293 cells. Transient transfection was achieved using Lipofectamine 2000 (Invitrogen) according to the manufacturer's instructions. Briefly, 2 µg channel plasmid and 1 µg QBI (QBiogene), which expresses GFP, was mixed with 3 µg Lipofectamine 2000 and added to each 35 mm dish. GFP expressing cells were identified by epi-fluorescence. Conventional whole cell recordings were made using an EPC9 amplifier controlled by Pulse software (Heka). Data were filtered at 1 kHz and acquired at 5 kHz. Borosilicate glass patch pipettes (2–5 MΩ) were coated with Sylgard resin, fire polished and filled with a solution containing (in mM): KCl 140, HEPES 10, K_2_HEDTA 5, and either 1.2 CaCl_2_ (free Ca^2+^  = 1 µM) or no added Ca^2+^ (free Ca^2+^ <10 nM). The pH was adjusted to 7.2 with 1 M KOH. Free Ca^2+^ concentrations were calculated using the REACT program (G.L. Smith, University of Glasgow) and stability constants for HEDTA published in Martell and Smith [19]. Except where stated the extracellular solution was the same as the incubation medium used in binding experiments. As with binding studies all experiments were performed at room temperature (20–25°C).

**Figure 1:**
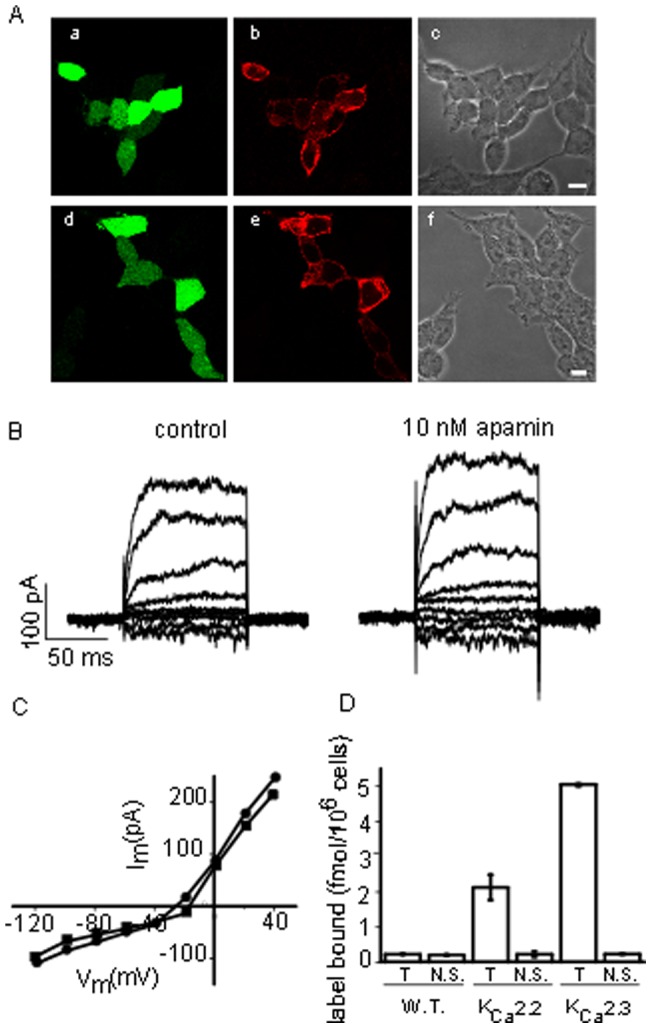
K_Ca_2 channels are not expressed in wild type HEK 293 cells. A Immunostaining of HEK 293 cells transiently transfected with GFP and K_Ca_2.2 (a,b,c) or K_Ca_2.3 (d,e,f). GFP expressing cells are visible in a and d. Comparison with the brightfield images in c and f shows that only a proportion of the cells were transfected. Staining for K_Ca_2 channels is shown in b and e where a signal is only visible in cells also expressing GFP. The scale bars indicate 10 µm. B Whole cell currents from a wild type HEK 293 cell recorded using a calcium containing internal solution before (▪) and after exposure to 10 nM apamin (•). The endogenous current is not inhibited. C Current voltage relationship for recordings in B. D Binding of ^125^I-apamin to wild type, K_Ca_2.2 and K_Ca_2.3 expressing HEK 293 cells. Cells were incubated with 30 pM ^125^I apamin in the absence (T) or presence (N.S.) of 100 nM UCL 1848. There is no significant binding in wild type cells. In contrast, total binding is much higher in cells expressing K_Ca_2 expressing cells and is almost completely inhibited by UCL 1848.

Routinely, cells were held at −80 mV and 100 ms test pulses applied to potentials between −120 mV and 40 mV. HEK 293 cells possess a small endogenous voltage-dependent outward current (see Fig. 1) which is activated at potentials positive to 0 mV. In order to avoid contamination of K_Ca_2 current by the endogenous currents, inhibition by blocking agents was measured at −20 mV. In practise it was found that under these cossnditions it was possible to obtain >90% inhibition of the current with K_Ca_2 channel blockers.

Concentration-inhibition curves were fitted by a variant of the Hill equation with the form:
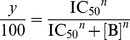
where *y* is the current in the presence of blocker at concentration [B] expressed as a percentage of control and *n* is the Hill coefficient.

### Immunohistochemistry

HEK 293 cells were plated on glass coverslips and transiently transfected with either K_Ca_2.2 and GFP or K_Ca_2.3 together with GFP, as described above. Cultures were then stained using rabbit polyclonal antibodies against K_Ca_2.2 or K_Ca_2.3 as previously described [20]. Briefly, cells were first washed in phosphate buffered saline (PBS; composition (mM): NaCl 136.9, KCl 2.7, Na_2_HPO_4_ 9.2, KH_2_PO_4_ 1.8, pH to 7.2 with HCl) and fixed in PBS containing 4% paraformaldehyde for 10 min. After rehydration in PBS the cells were permeabilised in methanol for 10 min followed by a 5 min wash in PBS. Next the cells were incubated in an antibody blocking solution (2% horse serum, 2% BSA in PBS) for 1 hr and then incubated in the appropriate primary antibody for 4 hr. The 4 hr incubation was followed by three washes in a PBS solution containing 1% Tween-20. The cells were then incubated in a solution containing a TRITC labelled goat anti-rabbit secondary antibody for 1 hr. The cells finally underwent three washes in PBS (containing 1% Tween-20) and the coverslips were mounted onto clean glass slides using an antifade mount (Vectashield, Vector Laboratories Incorporated).

### Materials

Rat K_Ca_2.2, subcloned into pTracer and a HEK 293 cell line stably expressing K_Ca_2.2 were kindly provided by Professor L.Kaczmarek, Yale University and Professor William Joiner, UCSD. UCL 1684 and UCL 1848 were prepared in the laboratory of Professor. C.R. Ganellin, UCL. Tissue culture reagents and Lipofectamine 2000 were purchased from Invitrogen. Apamin, gallamine, dequalinium, horse serum, bovine serum albumen and TRITC labelled goat anti-rabbit IgG were from Sigma. A stable HEK 293 K_Ca_2.3 cell line was created using zeocin selection following transfection with the rat K_Ca_2.3 subcloned into the pcDNA3.1 zeo plasmid (Invitrogen). [^125^I] mono-iodoapamin (^125^I-apamin) was supplied by New England Nuclear.

## Results

### Wild type HEK 293 cells do not express K_Ca_2 channels

In order to rule out the possibility that our results might be complicated by the endogenous expression of K_Ca_2 channels in HEK 293 cells, we performed a number of control experiments (Fig. 1). Firstly, we made patch-clamp recordings from wild type HEK cells in order to examine the endogenous currents. We saw no K_Ca_2-like (voltage-independent) currents but instead saw a small, voltage-dependent current. This endogenous current has been studied by Zhu *et al*. [21] who concluded that it was predominantly carried by chloride channels. It is therefore, perhaps not surprising that we found it could not be inhibited by 10 nM apamin (Fig. 1B, C). To further confirm our finding we stained cells transiently transfected with GFP and either K_Ca_2.2 or K_Ca_2.3. As is clear from Fig. 1A, antibody staining is visible only in transfected cells (i.e. those expressing GFP). Finally, we were unable to demonstrate any inhibitable binding of ^125^I-apamin to wild type HEK cells (Fig. 1D). Taken together, these observations show that there is no detectable expression of endogenous K_Ca_2 channels in the HEK293 cell line used in the present study.

### Binding of ^125^I-apamin to KCa2.2 channels in intact HEK 293 cells

**Figure 2:**
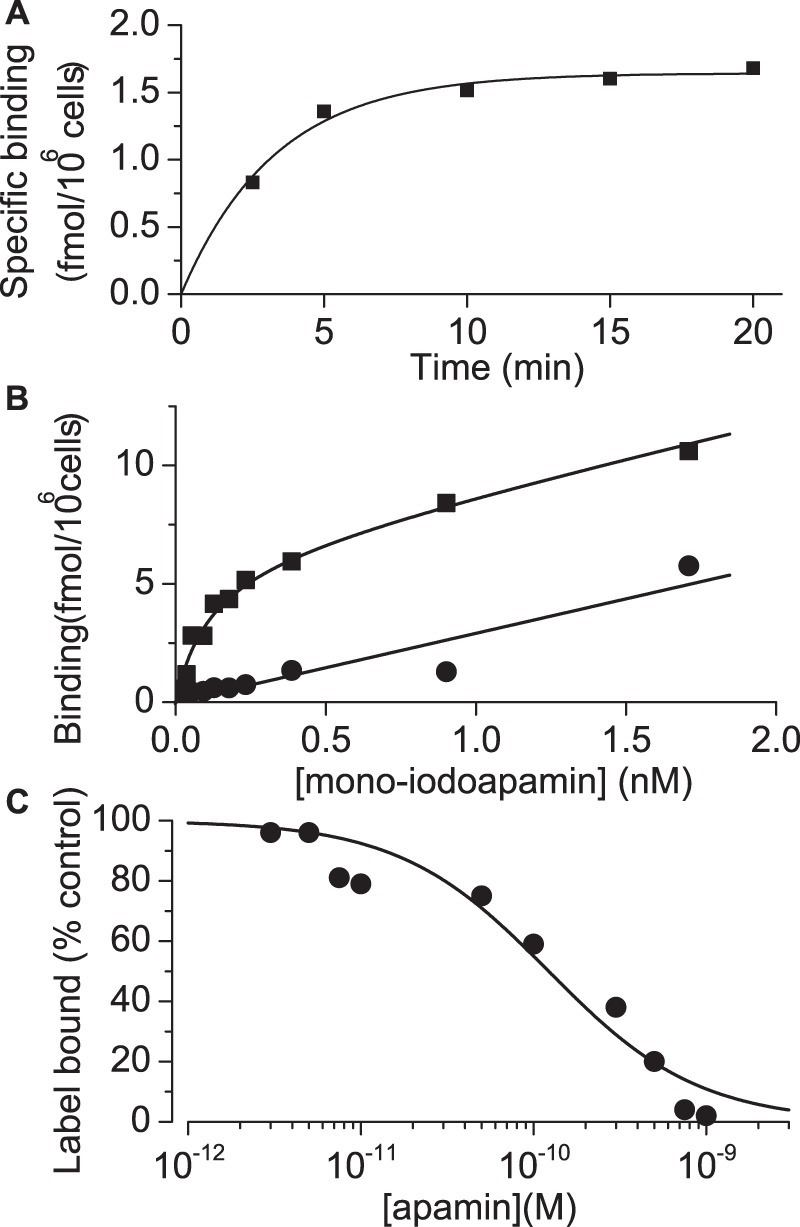
Binding of ^125^I-apamin to K_Ca_2.2. A Time course of ^125^I-apamin binding to HEK 293 cells stably expressing K_Ca_2.2. The *y*-axis shows specific binding of ^125^I-apamin at times indicated on the *x*-axis. Each point represents the mean of triplicate observations from a single experiment. The data are fitted by a single exponential function (solid line) with an apparent rate constant of 0.3±0.03 min^–1^_._ The mean rate constant from 3 separate experiments was 0.24±0.04 min^–1^ suggesting that binding reaches equilibrium well within 10 minutes. B Equilibrium binding of ^125^I-apamin to HEK 293 cells stably expressing K_Ca_2.2. The graph shows total (▪) and non-specific (•) binding in the presence of label. Each point is the mean of triplicate observations from a single experiment. Combined data yielded estimates of *K*_L_ and *B*_max_ of 91±40 pM and 6.4±1.3 fmol/10^6^ cells (n = 3). Solid lines represent a linear fit (non-specific binding) or a fit using the modified Hill equation for *B*_tot_ (see methods). C Inhibition of ^125^I-mono-iodoapamin binding to K_Ca_2.2 by unlabelled apamin. Each point is the mean of triplicate observations from a single experiment. Data were fitted to the Hill equation (solid line). Estimates of *K*_L_ from saturation binding experiments were used to estimate *K*_i_ as described in the methods section and the derived estimates are given in Table 1.

We began by studying the binding of ^125^I-apamin to K_Ca_2.2 expressed in HEK 293 cells suspended in a normal physiological buffer. In order to establish an appropriate incubation time for these experiments we first measured the kinetics of ^125^I-apamin association to K_Ca_2.2 channels. The binding curve (Fig. 2A) was well fitted by a single exponential, yielding an apparent rate constant of 0.3±0.03 min^–1^. Thus, to ensure that equilibrium was reached in subsequent binding experiments, we always incubated cells and ligand together for a minimum of 10 minutes. Fig. 2B shows results from a typical K_Ca_2.2 saturation binding experiment using this 10 minute incubation period. Fitting the Hill equation to pooled data from five experiments yielded estimates of 91±40 pM and 6.4±1.3 fmol/10^6^ cells for *K*_L_ and *B*_max_ respectively. The ability of unlabelled apamin to displace ^125^I-apamin was also tested in order to examine the possible influence of iodination on apamin binding. A typical example of the data obtained is shown in Fig. 2C. The value of *K*_i_ for unlabelled apamin obtained from competition studies was 103±2 pM, similar to the value of *K*_L_ for ^125^I-apamin. Thus, in these experiments, there appears to be little difference between apamin and ^125^I-apamin in terms of their ability to bind to the channels.

### Binding of ^125^I-apamin to KCa2.3 channels in intact HEK 293 cells

**Figure 3:**
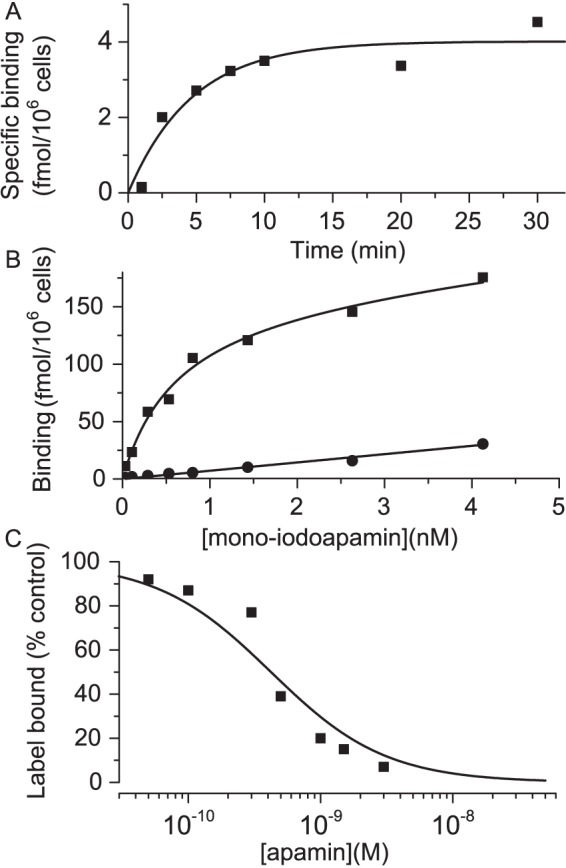
Binding of ^125^I-apamin to K_Ca_2.3. A Time course of ^125^I-apamin binding to HEK 293 cells stably expressing K_Ca_2.3. The *y* axis shows specific binding of ^125^I-apamin at times indicated on the *x*-axis. Each point represents the mean of triplicate observations from a single experiment. The data are fitted by a single exponential function (solid line) with a rate constant of 0.2±0.05 min^–1^_,_ suggesting binding reaches equilibrium within 10 minutes. B Equilibrium binding of ^125^I-apamin to HEK 293 cells stably expressing K_Ca_2.3. The graph shows total (▪) and non-specific (•) binding in the presence of label. Each point is the mean of triplicate observations from a single experiment. Combined data yielded estimates of *K*_L_ and *B*_max_ of 711±226 pM and 175±18 fmol/10^6^ cells. Solid lines represent a linear fit (non-specific binding) or a fit using the modified Hill equation for *B*_tot_ (see methods). C Inhibition of ^125^I-apamin binding to K_Ca_2.3 by unlabelled apamin. Each point is the mean of triplicate observations from a single experiment. Data were fitted to the Hill equation (solid line). Estimates of *K*_L_ from saturation binding experiments were used to estimate *K*_i_ as described in the methods section and the derived values are given in Table 1.

We next studied apamin binding to K_Ca_2.3 channels. Again we began by examining the kinetics of binding to ensure that appropriate incubation times were used. Figure 3A shows the results from one such experiment. Again the data fit reasonably well to a single exponential (with apparent rate constant of 0.2±0.05 min^–1^). Thus, again, a 10 minute incubation period was appropriate in order to ensure that equilibrium was reached. Data from our saturation binding experiments with K_Ca_2.3-expressing HEK cells are shown in Figure 3B. Fitting of these data provided values of 711±226 pM and 175±18 fmol/10^6^ cells for *K*_L_ and *B*_max_ respectively. This *K*_L_ value is significantly higher than the value we obtained for K_Ca_2.2 and of the same order as the IC_50_ reported for inhibition of K_Ca_2.3 current [11], [18]. Finally, we assessed the ability of unlabelled apamin to inhibit mono-iodoapamin binding and found a *K*_i_ value of 350±83 pM (Fig. 3C). This is lower than the estimate of *K*_L_ for ^125^I-apamin, but within a factor of approximately two, which may reflect either experimental variability or some modest effect of iodination on apamin binding.

### Inhibition of ^125^I-apamin binding by other K_Ca_2 channel modulators

**Figure 4:**
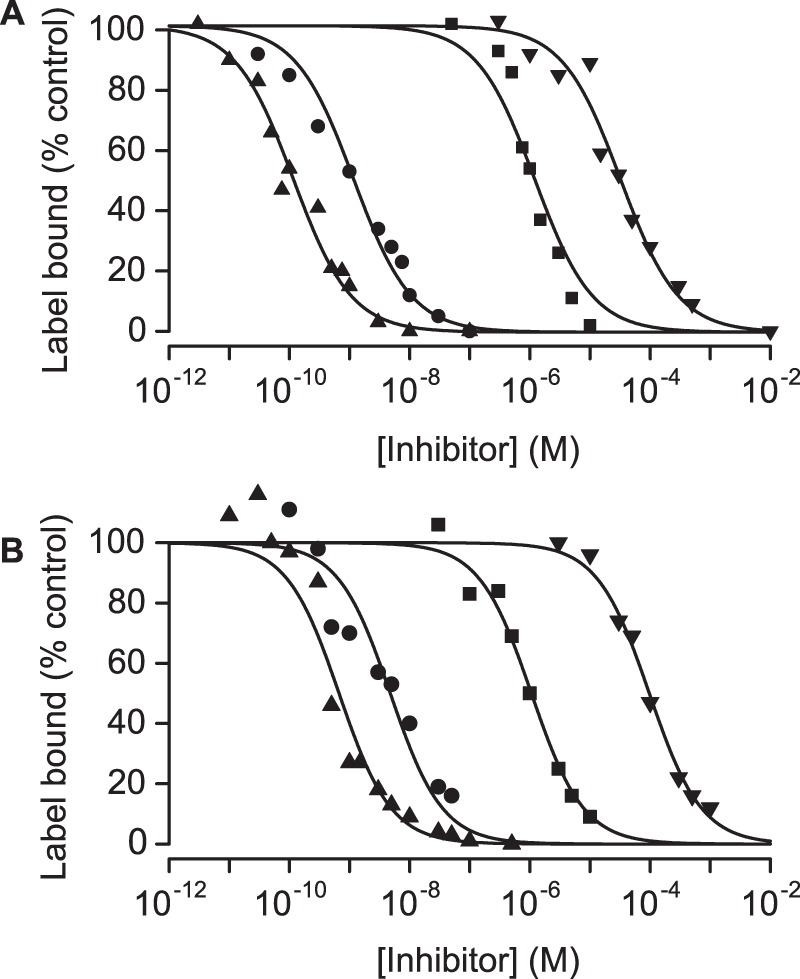
Inhibition of ^125^I-apamin binding to K_Ca_2.2 and 2.3 by known K_Ca_2 inhibitors. Inhibition of ^125^I-apamin binding to K_Ca_2.2(A) and K_Ca_2.3(B) by the known K_Ca_2 blockers UCL 1848 (▴), UCL 1684 (•), dequalinium (▪) and gallamine (▾). Each point is the mean of triplicate observations from a single experiment. Data were fitted to the Hill equation (solid lines) and the values of IC_50_ used to estimate *K*_i_ as described in Methods. The pooled data are shown in Table 1.

We next examined a range of small molecule blockers of K_Ca_2 channels using the ^125^I-apamin assay, since these have also been reported to have a variety of potencies and even to have different rank orders of selectivity in binding versus block experiments. We thus examined K_Ca_2.2 and then, in a separate experiment, K_Ca_2.3, testing inhibition of ^125^I-apamin binding by UCL 1848, UCL 1684, dequalinium and gallamine. Inhibition curves for these compounds are shown in Fig. 4A (K_Ca_2.2) and Fig. 4B (K_Ca_2.3). Data for inhibition of ^125^I-apamin binding could be described by an inhibition curve with a Hill slope of one. Further, within the margins of error for this experiment, it was clear that specific binding of apamin could be completely inhibited. The values of *K*_L_ estimated above were used to calculate estimates of *K*_i_ for the compounds tested which are summarized in Table 1. The rank order of potency, apamin ∼ UCL1848> UCL1684> dequalinium > gallamine was the same for both K_Ca_2.2 and K_Ca_2.3. However, all of the compounds showed selectivity for K_Ca_2.2 over K_Ca_2.3.

**Table 1:** Summary of IC_50_ for inhibition of K_Ca_2 current and *K*_i_ for displacement of mono-iodoapamin in cells expressing K_Ca_2.2 and K_Ca_2.3.

	K_Ca_2.2		K_Ca_2.3	
Compound	IC_50_	*K*_i_	IC_50_ a	*K*_i_
Apamin	112±1 pM	103±1.9 pM	1.4±0.2 nM	350±83 pM
UCL 1848	125±2.5 pM	95±1.7 pM	2.1±0.3 nM	652±180 pM
UCL1684	530±8 pM	1008±22 pM	5.8±0.3 nM	7.7±1.6 nM
Dequalinium	420±99 nM	977±165 nM	920±140 nM	865±160 nM
Gallamine	7±0.8 µM	26±5 µM	98±0.7 µM	71±8 µM

a Data from Hosseini *et al*. 2000, using CHO cells.

### Inhibition of K_Ca_2.2 current

**Figure 5:**
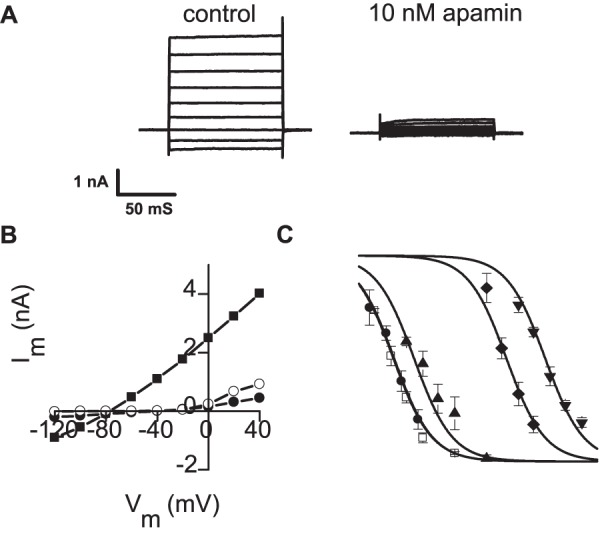
Effects of apamin and known K_Ca_2 inhibitors on K_Ca_2.2 current. A Typical current record from a HEK 293 cell transiently transfected with K_Ca_2.2. In control solution (left panel) robust, time-independent currents are seen. Application of 10 nM apamin causes a very substantial reduction (right panel). B Current voltage relationships for the cell in A in the absence (▪) and presence (•) of 10 nM apamin. In the absence of apamin the current reverses at −80 mV, close to the predicted value of *E*_K_. In the presence of apamin the residual current is similar to that seen in an untransfected HEK 293 cell (○) suggesting that at this concentration apamin causes nearly complete inhibition of K_Ca_2 current. C Concentration inhibition curves for apamin (•), UCL 1848 (□), UCL 1684 (▴), dequalinium (⧫) and gallamine (▾). Each point is the mean of 3–5 observations, vertical bars show s.e.m. Fitted lines were drawn from the Hill equation with a common Hill slope of 0.7±0.04 (see Methods). Note that the curves drawn for apamin and UCL 1848 overlie each other. At sufficiently high concentrations of blocker near complete inhibition of current is achieved. The IC_50_ values calculated for each compound are given in Table 1.

The same compounds used in binding studies were next assessed for their ability to inhibit K_Ca_2.2 current in a whole cell patch-clamp assay. We were careful to examine block of K_Ca_2.2 current under conditions that were essentially identical to those used in binding. As illustrated in Fig. 5A, HEK cells expressing K_Ca_2.2 exhibited robust, time-independent currents which reversed close to the predicted value of *E*_K_ (−85 mV) (Fig. 5A,C). In the presence of 10 nM apamin which, based on our binding experiments should be a near saturating concentration, this current was much reduced (Fig. 5A, right panel). Further, the residual current was time-dependent, showed weak outward rectification and reversed at −40 mV. Thus, following the addition of 10 nM apamin, the only remaining current appeared to be the endogenous current (not itself inhibitable by apamin) (Fig. 5B, Fig. 1C). Thus it appears that apamin causes near-complete block of K_Ca_2 current in our assay. Indeed, our results suggest that all of the compounds tested produced >90% inhibition of KCa2.2 current when applied at sufficiently high concentrations. IC_50_ values were obtained by fitting the Hill equation to the concentration-inhibition curves (Fig. 5C). The IC_50_ values are given in Table 1 for comparison with the *K*_i_ values obtained from binding. There is good agreement between the two datasets.

### The effect of changes in ionic composition on channel block

**Figure 6:**
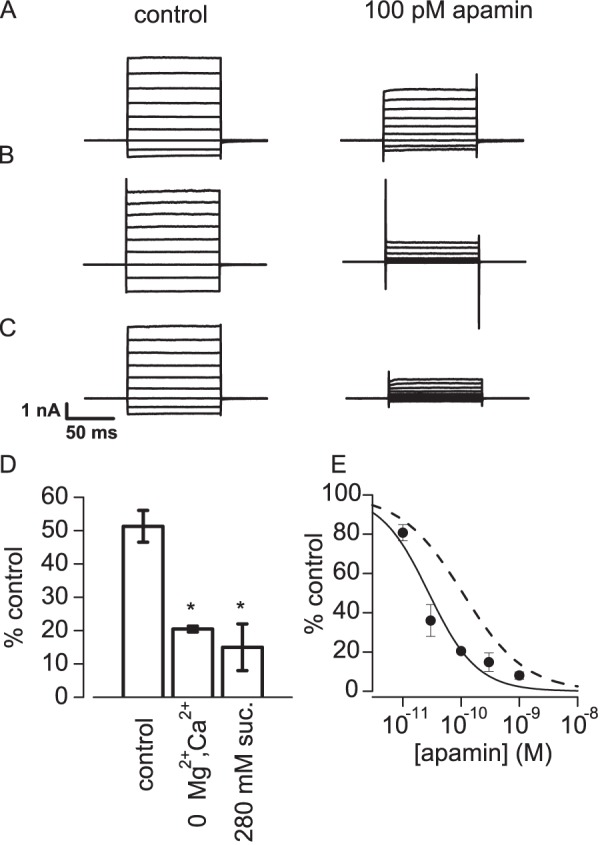
The effect of the ionic composition of bathing solution on the potency of apamin. Representative recordings of K_Ca_2 current in HEK 293 cells transiently transfected with K_Ca_2.2. The recordings were made in standard solution (A), nominally Ca ^2+^ and Mg^2+^ free solution (B) and Na^+^, Mg^2+^ and Ca^2+^ free solution in which NaCl was replaced by 280 mM sucrose (C). In each case the left panel shows currents recorded before and the right panel after the application of 100 pM apamin. D Summary of data for the inhibition caused by 100 pM apamin in the solutions used in A, B and C. Vertical bars show s.e.m. * denotes that the values were significantly different from the effect of apamin in standard solution (p<0.05, unpaired t-test). E Concentration-inhibition curve for apamin applied in Ca^2+^ and Mg^2+^ free solution. Each point is the mean of 3–5 observations with vertical bars indicating s.e.m. The solid line was drawn from a fit of the Hill equation with IC_50_ =  20±3 pM and *n* = 0.8±0.06. The dashed line represents the concentration-inhibition curve for apamin in standard solution shown in Figure 4.

Given the close correspondence between the potency of apamin measured by electrophysiological block and ligand binding assays in identical (physiological) solutions, it seemed important to understand why such disparate values have been reported in the literature. One obvious possibility concerns the different composition of solutions used in previously published binding studies. In binding experiments on isolated membrane preparations it is common to use solutions which have low ionic strength and are free of Ca^2+^ and Mg^2+^. We therefore examined the effect of changing our extracellular solution to mimic these differences (Fig. 6A,B,C,D). In our standard bath solution 100 pM reduced K_Ca_2.2 current to 51±5% of control. In contrast, when the bathing solution was free of added Ca^2+^ and Mg^2+^ 100 pM apamin reduced the K_Ca_2 current to 20±0.9% of control (p = <0.05 compared with standard). When, in addition to removal of Mg^2+^ and Ca^2+^, NaCl was replaced by 280 mM sucrose to mimic even more closely conditions reported in the literature, 100 pM apamin reduced K_Ca_2.2 current to 15±7% of control (p<0.05 compared with standard solution). The effect of removing Ca^2+^ and Mg^2+^ was further examined by constructing a concentration-inhibition curve for apamin in Mg^2+^ and Ca^2+^ free conditions (Fig. 6E). The IC_50_ and Hill slope were 20±3 pM and 0.8±0.06 respectively. Thus, simply removing divalent cations from the bath solution caused an approximately 5-fold increase in potency. These results suggest that the potency of apamin in binding to K_Ca_2.2 channels is increased under ionic conditions similar to those frequently used in binding assays. This may account for a substantial part of the very high affinity *K*_L_ and *K*_i_ values of apamin binding in previously published data.

## Discussion

### Binding and block for apamin

We find that when apamin binding and apamin block of K_Ca_2 channels are measured using the same ionic conditions, the values of *K*_i_ and *K*_L_ for binding and the value of IC_50_ for inhibition (block) of current agree well. Whilst previous reports in the literature found differences of up ∼20–200-fold, the largest discrepancy we found in comparing binding and block for both KCa2.2 and KCa2.3 values is a factor of only approximately four, and in that case the comparison is to IC_50_ values using a different cell line (CHO cells) in a study that was not part of the current work. Further, the IC_50_ values we observed are, overall, very similar to the values obtained by others using electrophysiological approaches to measure inhibition [11], [13], [15], [17], [22]. In contrast, previously published values of *K*_L_ for the binding of labelled apamin to membrane preparations of K_Ca_2.2-expressing HEK cells have been much lower, of the order of 6 pM [16], [17]. Moreover, whilst it is often reported that apamin is significantly more potent as a blocker of K_Ca_2.2 than K_Ca_2.3 (see e.g. Kohler *et al*.[11]) binding studies on isolated membranes suggest that apamin does not discriminate between the two sub-types [16], [17]. In the present study we found that labelled apamin bound to K_Ca_2.3 with a *K*_L_ of 711±226 pM and a *K*_i_ value of 350±83 pM. Even taking the lowest of these estimates the affinity of apamin is still significantly less for K_Ca_2.3 than K_Ca_2.2 (*K*_L_ 90±40 pM, *K*_i_ 103±2 pM). This is in keeping with IC_50_ values from functional experiments (see Table 1).

Other evidence supports our conclusion that the ionic conditions used in binding measurements markedly affect the outcome. For example, in intact guinea pig hepatocytes the *K*_L_ for apamin binding in normal physiological solutions was reported as 390 pM [6], of the same order as the concentration needed to cause 50% block in functional experiments. In marked contrast, a value of 3 pM was reported for binding to hepatocyte membranes, an experiment performed in low ionic strength [23]. Similarly, in primary cultures of intact cortical neurones a value of 60 to 120 pM was reported by Seagar *et al*. [24] in normal physiological solutions, compared with a value of 6 pM for isolated cortical membranes, again at low ionic strength [16]. Finally, Hugues *et al*. [5] reported a similar discrepancy between binding of labelled apamin to, and inhibition of, the apamin-sensitive AHP in NIE115 neuroblastoma cells. Further work by this group showed that the binding of labelled apamin was inhibited by divalent cations including Mg^2+^ and Ca^2+^ and by Na^+^. In keeping with this observation we have now demonstrated that removal of Ca^2+^ and Mg^2+^ caused an increase in the blocking action of apamin. In experiments on isolated membranes it is standard practice to use a low ionic strength solution (e.g., 10 mM Tris, 5.4 mM KCl, pH 7.5 as employed by Lamy *et al*. [17]. Taken together, the evidence now available suggests that ionic environment is highly important in determining the affinity of apamin for its binding site. Whether this is due to charge screening or other changes to the channel-toxin interaction remains to be determined. Nor is it possible to exclude the possibility that the conformation and charge distribution in apamin itself changes in a low ionic strength solution devoid of Ca^2+^, Mg^2+^ and Na^+^. It is interesting in relation to this to note that binding/action discrepancy seen under such experimental conditions is not observed with all blockers e.g. UCL 1684 [25], even though this compound carries two positive charges to mimic the charges that are thought to be important in apamin binding.

It is also interesting to compare the values of *K*_L_ for the binding of apamin obtained in this study with values for the IC_50_ obtained in preparations natively expressing K_Ca_2 channels of known subtype. K_Ca_2.2 has been shown to underlie the Ca^2+^-activated K^+^ current seen in Jurkat T cells [26] and these native channels are blocked by apamin with an IC_50_ of 300 pM [27], a value that is much higher than would be expected from membrane binding experiments performed in non-physiological ionic conditions. Similarly, rat sympathetic ganglion neurones and dorsal vagal neurones display a post-spike after-hyperpolarisation which is known to be mediated by K_Ca_2.3 and is inhibited by apamin with an IC_50_ of ∼2 nM [18], [28], again a value that is much higher than expected from binding studies of isolated membranes. However, in both of these cases the values are similar to those obtained from functional studies of heterologously expressed channels and from the values of *K*_L_ obtained in binding experiments (using physiological solutions) in the present study. Further, looking at the different values for native K_Ca_2.2 and K_Ca_2.3 channels it appears that subtype selectivity is also observed in this setting and may thus be useful for elucidating the function of KCa2 channels in the future.

### Investigation of K_Ca_2 pharmacology by ligand binding and electrophysiology

We also compared the potency of some known K_Ca_2 channel blockers in electrophysiological and binding inhibition experiments. With cells expressing K_Ca_2.2 there was good agreement between *K*_i_ and IC_50_ values for all the compounds tested (UCL1848, UCL 1684, dequalinium and gallamine; Table 1). The largest difference between *K*_i_ and IC_50_ was a factor 3.7 for gallamine, the least active of the set and therefore the most likely to show non-specific actions. Similar agreement was seen with K_Ca_2.3 (Table 1). The implication is that provided the binding studies are done using bathing fluids with ionic composition not far removed from physiological, they can give a good prediction of biological activity.

### Implications for interpreting the mechanism of apamin block of K_Ca_2 channels

Early experiments suggested that apamin might simply act as a pore blocker for K_Ca_2 channels because TEA (a known small molecule pore blocker), prevented the binding of apamin [16], [29]. Nonetheless, mutation studies have shown that interactions of apamin with the channel involve a number of amino acid residues, some of which are outside the central pore region [22], [30], [31]. This and other evidence has led to the suggestion that apamin does not act as a ‘classical’ pore blocker but instead inhibits channel opening by an allosteric mechanism [17], [32]. The discrepancy between the *K*_L_ for binding of apamin (measured in low ionic strength) and IC_50_ for inhibition of K_Ca_2.2 current (measured in normal physiological solutions) has been cited as evidence in support of this idea. Our findings suggest that caution must be exercised when making this kind of comparison. In particular, it is clear that ligand binding experiments agree much more closely with functional experiments when conducted under identical physiological conditions. Of course, agreement between IC_50_ and *K*_i_ values does not exclude an allosteric mode of action and indeed it is quite difficult to imagine an allosteric mechanism that does not predict close accord in the concentration dependence of both binding and block (otherwise block would still be able to increase when binding had already saturated). On the other hand, some allosteric mechanisms would predict incomplete block of current even at high concentrations of apamin. We see no clear evidence of this, in keeping with a number of other reports [11], [13], [22]. Nonetheless, two groups do report partial block [17], [33] so that more work is needed to clarify this aspect of the action of apamin.

In summary, we have found that there is a close relationship between the binding and block of K_Ca_2 channels for both apamin and other small molecule blockers when these are measured under identical conditions. While this observation is consistent with both allosteric and non-allosteric models it suggests that caution must be exercised when comparing electrophysiological block data obtained in normal (physiological) conditions with binding data obtained in non-physiological conditions. Our findings also suggest that more work may be needed to establish precisely the mechanism of action of apamin and other compounds known to block K_Ca_2 channels.
